# The spectral properties of $[m]$-complex symmetric operators

**DOI:** 10.1186/s13660-018-1800-1

**Published:** 2018-08-14

**Authors:** Junli Shen

**Affiliations:** 1 College of Computer and Information Technology, Henan Normal University, Xinxiang, China; 2 College of Mathematics and Information Sciences, Shaanxi Normal University, Xi’an, China

**Keywords:** 47B20, 47A05, $[m]$-complex symmetric operator, Property (*β*), Nilpotent operator

## Abstract

In this paper, we introduce the class of [*m*]-complex symmetric operators and study various properties of this class. In particular, we show that if *T* is an [*m*]-complex symmetric operator, then $T^{n}$ is also an [*m*]-complex symmetric operator for any $n\in\mathbb {N}$. In addition, we prove that if *T* is an [*m*]-complex symmetric operator, then $\sigma_{a}(T)$, $\sigma_{\mathrm{SVEP}}(T)$, $\sigma_{\beta }(T)$, and $\sigma_{(\beta)_{\epsilon}}(T)$ are symmetric about the real axis. Finally, we investigate the stability of an [*m*]-complex symmetric operator under perturbation by nilpotent operators commuting with *T*.

## Introduction

Let *H* be a complex separable Hilbert space, and let $B(H)$ denote the algebra of all bounded linear operators on *H*. For $T\in B(H)$, we denote the approximate point spectrum of *T* by $\sigma_{a}(T)$.

Let *A* and *B* be given operators in $B(H)$. Recall the definition of the usual derivation operator $\delta_{A,B}(X)$ given by $\delta_{A,B}(X)= AX-XB$ for $X\in B(H)$. For every positive integer *k*, we have $\delta^{k}_{A,B}(X)= \delta_{A,B}(\delta^{k-1}_{A,B}(X))$ for $X\in B(H)$. Let *A* and *B* be in $B(H)$. An operator *B* is said to be in $\mathrm{Helton}_{k}(A)$ if $\delta^{k}_{A,B}(I)=0$.

In operator theory, one of the most important topics is local spectral theory. In the following we consider several properties in local spectral theory such as the single-valued extension property, property (*β*), property $(\beta )\epsilon$, and so on. Let $D(\lambda,r)$ be the open disc centered at $\lambda\in\mathbb {C}$ and with radius $r > 0$. For an open set *U* in $\mathbb {C}$, we denote by $\mathcal{O}(U, H)$ and $\mathcal{\xi}(U, H)$ the Fréchet space of all *H*-valued analytic functions on *U* and the Fréchet space of all *H*-valued $C^{\infty}$-functions on *U*, respectively.

An operator $T\in B(H)$ is said to have the single-valued extension property (SVEP for short) at $\lambda_{0}\in\mathbb {C}$ if, for every open neighborhood *G* of $\lambda_{0}$, the only analytic function $f: G\rightarrow H$ which satisfies the equation $(\lambda I-T)f(\lambda)=0$ for all $\lambda\in G$ is the function $f\equiv0$. An operator *T* is said to have SVEP if *T* has SVEP at every point $\lambda\in\mathbb {C}$. An operator $T\in B(H)$ is said to satisfy Bishop’s property (*β*) at $\lambda_{0}\in\mathbb {C}$ (resp. $(\beta)\epsilon$) if there exists $r>0$ such that, for every open subset $U\subset D(\lambda,r)$ and for any sequence $(f_{n})$ in $\mathcal{O}(U, H)$ (resp. in $\mathcal{\xi }(U, H)$), whenever $(T - z)f_{n}(z)\rightarrow0$ in $\mathcal{O}(U, H)$ (resp. in $\mathcal{\xi}(U, H)$), then $f_{n}\rightarrow0$ in $\mathcal{O}(U, H)$ (resp. in $\mathcal{\xi}(U, H)$). An operator *T* is said to have Bishop’s property (*β*) (resp. $(\beta )\epsilon$) if *T* has Bishop’s property (*β*) (resp. $(\beta)\epsilon$) at every point $\lambda\in\mathbb {C}$.

Define

$$\begin{aligned} &\sigma_{\mathrm{SVEP}}(T)=\{\lambda\in\mathbb {C}:T-\lambda\mbox{ fails to SVEP at } \lambda\}; \\ &\sigma_{\beta}(T)= \bigl\{ \lambda\in\mathbb {C}:T-\lambda\mbox{ fails to property } (\beta) \mbox{ at } \lambda \bigr\} ; \\ &\sigma_{(\beta)_{\epsilon}}(T)= \bigl\{ \lambda\in\mathbb {C}:T-\lambda \mbox{ fails to property } (\beta)_{\epsilon} \mbox{ at } \lambda \bigr\} . \end{aligned}$$

An antilinear operator *C* on *H* is said to be conjugation if *C* satisfies $C^{2} = I$ and $(Cx,Cy)=(y,x)$ for all $x,y\in H$. An operator $T\in B(H)$ is said to be complex symmetric if $T^{*}=CTC$. Many standard operators such as normal operators, algebraic operators of order 2, Hankel matrices, finite Toeplitz matrices, all truncated Toeplitz operators, and Volterra integration operators are included in the class of complex symmetric operators. Several authors have studied the structure of a complex symmetric operator. We refer the reader to [6–11] for further details. As a generalization of complex symmetric operators, in [3], Chō et al. introduced *m*-complex symmetric operators with conjugation *C* as follows: For an operator $T\in B(H)$ and an integer $m\geq1$, *T* is said to be an *m*-complex symmetric operator if there exists some conjugation *C* such that

$$\sum_{j=0}^{m}(-1)^{m-j} \begin{pmatrix}m\\j\end{pmatrix}T^{*j}.CT^{m-j}C=0. $$

In 1990s, Agler and Stankus [1] intensively studied the following operator: For a fixed positive integer *m*, an operator $T\in B(H)$ is said to be an *m*-isometric operator if it satisfies the following equation:

$$\sum_{j=0}^{m}(-1)^{j} \begin{pmatrix}m\\j\end{pmatrix}T^{*m-j}T^{m-j}=0. $$

*m*-isometric operators are connected to Toeplitz operators, classical function theory, ordinary differential equations, distributions, classical conjugate point theory, Fejer–Riesz factorization, stochastic processes, and other topics.

In [4], Chō et al. introduced ($m,C$)-isometric operators with conjugation *C* as follows: For an operator $T\in B(H)$ and an integer $m\geq1$, *T* is said to be an ($m,C$)-isometric operator if there exists some conjugation *C* such that

$$\sum_{j=0}^{m}(-1)^{j} \begin{pmatrix}m\\j\end{pmatrix}T^{*m-j}.CT^{m-j}C=0. $$

In [5], Chō et al. introduced [$m,C$]-isometric operators with conjugation *C* as follows: For an operator $T\in B(H)$ and an integer $m\geq1$, *T* is said to be an [$m,C$]-isometric operator if there exists some conjugation *C* such that

$$\sum_{j=0}^{m}(-1)^{j} \begin{pmatrix}m\\j\end{pmatrix}CT^{m-j}C.T^{m-j}=0. $$

According to the definitions of complex symmetric, *m*-complex symmetric, *m*-isometric, ($m,C$)-isometry, and [$m,C$]-isometry, we define [*m*]-complex symmetric *T* as follows: An operator *T* is said to be an [*m*]-complex symmetric operator if there exists some conjugation *C* such that

$$\sum_{j=0}^{m}(-1)^{m-j} \begin{pmatrix}m\\j\end{pmatrix}CT^{j}C.T^{m-j}=0. $$

For an operator $T\in B(H)$ and a conjugation *C*, we define the operator $w_{m}(T,C)$ by

$$w_{m}(T,C)=\sum_{j=0}^{m}(-1)^{m-j} \begin{pmatrix}m\\j\end{pmatrix}CT^{j}C.T^{m-j}. $$

Then *T* is an [*m*]-complex symmetric operator if and only if $w_{m}(T,C)= 0$. Moreover, it holds that

$$CTC.w_{m}(T,C)-w_{m}(T,C).T= w_{m+1}(T,C). $$

Hence if *T* is an [*m*]-complex symmetric operator, then *T* is an [*n*]-complex symmetric operator for every $n\geq m$.

The following example provides an operator which is a [3]-complex symmetric operator but not a [2]-complex symmetric operator.

### Example 1.1

Let $H=\mathbb {C}^{2}$, and let *C* be a conjugation on *H* given by $C(x, y) = (\overline{y}, \overline{x})$. If $T= \bigl ( {\scriptsize\begin{matrix}{} 0&1\cr 0&0 \end{matrix}} \bigr )$ on $\mathbb {C}^{2}$, we have $CTC= \bigl ( {\scriptsize\begin{matrix}{} 0&0\cr 1&0 \end{matrix}} \bigr )$, then

$$\begin{aligned} &(CTC)^{2}-2CTC.T+T^{2} \\ &\quad=\left ( \begin{matrix} 0&0\\ 1&0 \end{matrix} \right )^{2}-2\left ( \begin{matrix} 0&0\\ 1&0 \end{matrix} \right )\left ( \begin{matrix} 0&1\\ 0&0 \end{matrix} \right )+\left ( \begin{matrix} 0&1\\ 0&0 \end{matrix} \right )^{2} \\ &\quad=\left ( \begin{matrix} 0&0\\ 0&-2 \end{matrix} \right ). \end{aligned}$$

Hence *T* is not a [2]-complex symmetric operator.

On the other hand, since

$$\begin{aligned} &(CTC)^{3}-3(CTC)^{2}.T+3(CTC).T^{2}-T^{3} \\ &\quad =\left ( \begin{matrix} 0&0\\ 1&0 \end{matrix} \right )^{3}-3\left ( \begin{matrix} 0&0\\ 1&0 \end{matrix} \right )^{2}\left ( \begin{matrix} 0&1\\ 0&0 \end{matrix} \right ) \\ &\qquad{}+3\left ( \begin{matrix} 0&0\\ 1&0 \end{matrix} \right )\left ( \begin{matrix} 0&1\\ 0&0 \end{matrix} \right )^{2}-\left ( \begin{matrix} 0&1\\ 0&0 \end{matrix} \right )^{3} \\ &\quad =0. \end{aligned}$$

Hence *T* is a [3]-complex symmetric operator.

### Example 1.2

Let $T\in B(H)$ and *C* be a conjugation on *H*. If *T* is nilpotent of order *k*, then *T* is a [$2k-1$]-complex symmetric operator with conjugation *C*. Indeed, since *T* is nilpotent of order *k*, it gives that $CT^{j}C=T^{j}=0$ for all $j\geq k$. Then since $\operatorname{max}\{j,2k-1-j\}\geq k$ for any $j \ (j=0,1,2,\ldots,2k-1)$, we get

$$\sum_{j=0}^{2k-1}(-1)^{2k-1-j} \begin{pmatrix}2k-1\\j\end{pmatrix}CT^{j}C.T^{2k-1-j}=0. $$

Hence *T* is a [$2k-1$]-complex symmetric operator with conjugation *C*.

### Example 1.3

Let *C* be a conjugation on *H* and $R\in B(H)$ satisfy $R=CRC$. If $RQ=QR$ and $Q^{k}=0$ for some *k*, then $T=R+Q$ is a [$2k-1$]-complex symmetric operator with conjugation *C*.

Indeed, we will show that $w_{m}(T,C)=w_{m}(Q,C)$ for any $m\in\mathbb {N}$. It is clear if $m=1$. Suppose that

$$\begin{aligned} w_{m-1}(T,C)={}&\sum_{j=0}^{m-1}(-1)^{m-1-j} \begin{pmatrix}m-1\\j\end{pmatrix}CT^{j}C.T^{m-1-j} \\ ={}&\sum_{j=0}^{m-1}(-1)^{m-1-j} \begin{pmatrix}m-1\\j\end{pmatrix}CQ^{j}C.Q^{m-1-j} \\ ={}&w_{m-1}(Q,C). \end{aligned}$$

Then, since $CRC=R$ and *R* commutes with $w_{m-1}(T)$, we have

$$\begin{aligned} w_{m}(T,C)={}&\sum_{j=0}^{m}(-1)^{m-j} \begin{pmatrix}m\\j\end{pmatrix}CT^{j}C.T^{m-j} \\ ={}&CTC \left[\sum_{j=0}^{m-1}(-1)^{m-1-j} \begin{pmatrix}m-1\\j\end{pmatrix}CT^{j}C.T^{m-1-j} \right] \\ &{}- \left[\sum_{j=0}^{m-1}(-1)^{m-1-j} \begin{pmatrix}m-1\\j\end{pmatrix}CT^{j}C.T^{m-1-j} \right]T \\ ={}&CQC \left[\sum_{j=0}^{m-1}(-1)^{m-1-j} \begin{pmatrix}m-1\\j\end{pmatrix}CQ^{j}C.Q^{m-1-j} \right] \\ &{}- \left[\sum_{j=0}^{m-1}(-1)^{m-1-j} \begin{pmatrix}m-1\\j\end{pmatrix}CQ^{j}C.Q^{m-1-j} \right]Q \\ ={}&\sum_{j=0}^{m}(-1)^{m-j} \begin{pmatrix}m\\j\end{pmatrix}CQ^{j}C.Q^{m-j} \\ ={}&w_{m}(Q,C). \end{aligned}$$

Since *Q* is a [$2k-1$]-complex symmetric operator, i.e.,

$$\sum_{j=0}^{2k-1}(-1)^{2k-1-j} \begin{pmatrix}2k-1\\j\end{pmatrix}CQ^{j}C.Q^{2k-1-j}=0. $$

Then $T=R+Q$ is a [$2k-1$]-complex symmetric operator with conjugation *C*.

## [*m*]-complex symmetric operators

### Theorem 2.1

*Let* $T\in B(H)$ *be an* [*m*]-*complex symmetric operator*. *If* $\lambda\in\sigma_{a}(T)$, *then* $\overline{\lambda}\in\sigma _{a}(T)$. *In particular*, *if* *λ* *is an eigenvalue of* *T*, *then* *λ̅* *is also an eigenvalue of* *T*.

### Proof

Let $\{x_{n}\}$ be a sequence of unit vectors such that $\lim_{n\rightarrow\infty}(T-\lambda)x_{n} = 0$. Since *T* is an [*m*]-complex symmetric operator and $\lim_{n\rightarrow\infty}(T^{k}-\lambda^{k})x_{n} = 0$ for all $k\in\mathbb {N}$, it follows that

$$\begin{aligned} 0={}&\lim_{n\rightarrow\infty} \left(\sum_{j=0}^{m}(-1)^{m-j} \begin{pmatrix}m\\j\end{pmatrix}CT^{j}C.T^{m-j}x_{n} \right) \\ ={}& C\lim_{n\rightarrow\infty} \left(\sum_{j=0}^{m}(-1)^{m-j} \begin{pmatrix}m\\j\end{pmatrix}T^{j}\overline{ \lambda}^{m-j} \right)Cx_{n} \\ ={}& C\lim_{n\rightarrow\infty}(T-\overline{\lambda})^{m}Cx_{n}. \end{aligned}$$

Moreover, since $C^{2}=I$, it follows that $\lim_{n\rightarrow\infty}(T-\overline{\lambda})^{m}Cx_{n}=0$. Since $\Vert Cx_{n} \Vert =1$, hence $\overline{\lambda}\in\sigma_{a}(T)$. □

### Theorem 2.2

*Let* $T\in B(H)$ *be an* [*m*]-*complex symmetric operator*. *If* *T* *has property* $(\beta)\epsilon$ *at* *λ*, *then* *T* *has property* $(\beta)\epsilon$ *at* *λ̅*. *In particular*, *if* *T* *has property* $(\beta)$ *at* *λ*, *then* *T* *has property* $(\beta)$ *at* *λ̅*, *and if* *T* *has SVEP at* *λ*, *then* *T* *has SVEP at* *λ̅*.

### Proof

If *T* has property $(\beta)\epsilon$ at *λ*, then $CTC$ has property $(\beta)\epsilon$ at *λ̅* by [2, Theorem 4.3]. Suppose that *T* is an [*m*]-complex symmetric operator. Then

$$\sum_{j=0}^{m}(-1)^{m-j} \begin{pmatrix}m\\j\end{pmatrix}CT^{j}C.T^{m-j}=0. $$

It follows that $T\in \mathrm{Helton}_{m}(CTC)$. Hence *T* has property $(\beta)\epsilon$ at *λ̅* by [2, Theorem 5.3]. □

We investigate the power $T^{n}$ and the inverse $T^{-1}$ of an [*m*]-complex symmetric operator *T* and show that the class of [*m*]-complex symmetric operators is norm closed.

### Theorem 2.3

*Let* *C* *be a conjugation on* *H*, *and let* $T\in B(H)$. *Then the following assertions hold*.

(i) *If* *T* *is invertible*, *then* *T* *is an* [*m*]-*complex symmetric operator if and only if so is* $T^{-1}$.
(ii) *If* *T* *is an* [*m*]-*complex symmetric operator*, *then* $T^{n}$ *is also an* [*m*]-*complex symmetric operator for any* $n\in\mathbb {N}$.
(iii) *If* $\{T_{n}\}$ *is a sequence of* [*m*]-*complex symmetric operators such that* $\lim_{n\rightarrow\infty} \Vert T_{n}-T \Vert =0$, *then* *T* *is also an* [*m*]-*complex symmetric operator*.

### Proof

(i) Suppose that *T* is an invertible [*m*]-complex symmetric operator. Since $C^{2}=I$, it follows that

$$\begin{aligned} 0={}& \bigl(CT^{-m}C \bigr) \left[\sum_{j=0}^{m}(-1)^{m-j} \begin{pmatrix}m\\j\end{pmatrix}CT^{j}C.T^{m-j} \right]T^{-m} \\ ={}&\sum_{j=0}^{m}(-1)^{m-j} \begin{pmatrix}m\\j\end{pmatrix}C \bigl(T^{-1} \bigr)^{m-j}C.T^{-(j)}, \end{aligned}$$

we have $\sum_{j=0}^{m}(-1)^{m-j}\bigl ({\scriptsize\begin{matrix}{}m\cr j\end{matrix}} \bigr )C(T^{-1})^{j}C.T^{-(m-j)}=0$. Hence $T^{-1}$ is also an [*m*]-complex symmetric operator.

(ii) Since

$$\begin{aligned} \bigl(a^{n}-b^{n} \bigr)^{m}={}&(a-b)^{m} \bigl(a^{n-1}+a^{n-2}b+a^{n-3}b^{2}+\cdots +b^{n-1} \bigr)^{m} \\ ={}&(a-b)^{m} \bigl(f_{0}a^{m(n-1)} +f_{1}a^{m(n-1)-1}b + f_{2}a^{m(n-1)-2}b^{2} \\ &{}+\cdots+ f_{m(n-1)}b^{m(n-1)} \bigr), \end{aligned}$$

where $f_{i}$, $i = 0,1,2,\ldots,m(n-1)$ are coefficients, it follows that

2.1 $$\begin{aligned} w_{m} \bigl(T^{n},C \bigr)=\sum _{i=0}^{m(n-1)}f_{i}CT^{m(n-1)-i}Cw_{m}(T,C)T^{i}. \end{aligned}$$

From (2.1), if $w_{m}(T,C)= 0$, then $w_{m}(T^{n},C)= 0$. Hence $T^{n}$ is an [*m*]-complex symmetric operator for any $n\in\mathbb {N}$.

(iii) Suppose that $\{T_{n}\}$ is a sequence of [*m*]-complex symmetric operators such that $\lim_{n\rightarrow\infty} \Vert T_{n}-T \Vert =0$. Then

$$\begin{aligned} & \left \Vert \sum_{j=0}^{m}(-1)^{m-j} \begin{pmatrix}m\\j\end{pmatrix}CT_{n}^{j}C.T_{n}^{m-j}- \sum_{j=0}^{m}(-1)^{m-j} \begin{pmatrix}m\\j\end{pmatrix}CT^{j}C.T^{m-j} \right \Vert \\ &\quad\leq \left \Vert \sum_{j=0}^{m}(-1)^{m-j} \begin{pmatrix}m\\j\end{pmatrix}CT_{n}^{j}C.T_{n}^{m-j}- \sum_{j=0}^{m}(-1)^{m-j} \begin{pmatrix}m\\j\end{pmatrix}CT_{n}^{j}C.T^{m-j} \right \Vert \\ &\qquad{}+ \left \Vert \sum_{j=0}^{m}(-1)^{m-j} \begin{pmatrix}m\\j\end{pmatrix}CT_{n}^{j}C.T^{m-j}- \sum _{j=0}^{m}(-1)^{m-j} \begin{pmatrix}m\\j\end{pmatrix}CT^{j}C.T^{m-j} \right \Vert \\ &\quad \leq\sum_{j=0}^{m} \begin{pmatrix}m\\j\end{pmatrix} \bigl\Vert CT_{n}^{j}C \bigr\Vert \bigl\Vert T_{n}^{m-j}-T^{m-j} \bigr\Vert +\sum _{j=0}^{m}\begin{pmatrix}m\\j\end{pmatrix} \bigl\Vert CT_{n}^{j}C-CT^{j}C \bigr\Vert \bigl\Vert T^{m-j} \bigr\Vert \\ &\quad \leq\sum_{j=0}^{m} \begin{pmatrix}m\\j\end{pmatrix} \Vert CT_{n}C \Vert ^{j} \Vert T_{n}-T \Vert \Biggl(\sum_{i=0}^{m-j-1} \Vert T_{n} \Vert ^{m-j-1-i} \Vert T \Vert ^{i} \Biggr) \\ &\qquad{}+\sum_{j=0}^{m} \begin{pmatrix}m\\j\end{pmatrix} \Vert CT_{n}C-CTC \Vert \Biggl(\sum _{i=0}^{j-1} \Vert CT_{n}C \Vert ^{j-1-i} \Vert T \Vert ^{i} \Biggr) \Vert T \Vert ^{m-j} \\ &\quad \leq\sum_{j=0}^{m} \begin{pmatrix}m\\j\end{pmatrix} \Vert T_{n} \Vert ^{j} \Vert T_{n}-T \Vert \Biggl(\sum_{i=0}^{m-j-1} \Vert T_{n} \Vert ^{m-j-1-i} \Vert T \Vert ^{i} \Biggr) \\ &\qquad{}+\sum_{j=0}^{m} \begin{pmatrix}m\\j\end{pmatrix} \Vert T_{n}-T \Vert \Biggl(\sum _{i=0}^{j-1} \Vert CT_{n}C \Vert ^{j-1-i} \Vert T \Vert ^{i} \Biggr) \Vert T \Vert ^{m-j}\rightarrow0. \end{aligned}$$

Since $\{T_{n}\}$ is an [*m*]-complex symmetric operator with conjugation *C*,

$$\sum_{j=0}^{m}(-1)^{m-j} \begin{pmatrix}m\\j\end{pmatrix}CT_{n}^{j}C.T_{n}^{m-j}=0, $$

we have

$$\sum_{j=0}^{m}(-1)^{m-j} \begin{pmatrix}m\\j\end{pmatrix}CT^{j}C.T^{m-j}=0, $$

i.e., *T* is an [*m*]-complex symmetric operator. □

We examine the nilpotent perturbations of an [*m*]-complex symmetric operator.

### Theorem 2.4

*Let* *T* *be an* [*m*]-*complex symmetric operator with conjugation* *C* *and* *N* *be a nilpotent operator of order* *n* *such that* $TN=NT$. *Then* $T+N$ *is an* [$m+2n-2$]-*complex symmetric operator with conjugation* *C*.

### Proof

First we prove

2.2 $$\begin{aligned} w_{m}(T+N,C)=\sum_{i+j+k=m} \begin{pmatrix}m\\i,j,k\end{pmatrix}CN^{j}C.w_{k}(T,C).(-N)^{i}, \end{aligned}$$

where $\bigl ({\scriptsize\begin{matrix}{}{m}\cr {i,j,k}\end{matrix}} \bigr )=\frac{m!}{i!.j!,k!}$ and $\lambda_{0}(*)=I$. It is easy to show that (2.2) holds for $m=1$. Assume that (2.2) holds for *m*, next we should prove (2.2) holds for $m+1$. Since

$$\begin{aligned} w_{m+1}(T+N,C)={}&C(T+N)C.w_{m}(T+N,C)-w_{m}(T+N,C).(T+N) \\ ={}&C(T+N)C.\sum_{i+j+k=m}\begin{pmatrix}m\\i,j,k\end{pmatrix}CN^{j}C.w_{k}(T,C).(-N)^{i} \\ &{}-\sum_{i+j+k=m}\begin{pmatrix}m\\i,j,k\end{pmatrix}CN^{j}C.w_{k}(T,C).(-N)^{i}(T+N) \\ ={}&\sum_{i+j+k=m}\begin{pmatrix}m\\i,j,k\end{pmatrix}CN^{j}C \bigl[CTC.w_{k}(T,C)-w_{k}(T,C).T \bigr].(-N)^{i} \\ &{}+\sum_{i+j+k=m}\begin{pmatrix}m\\i,j,k\end{pmatrix}CN^{j+1}C.w_{k}(T,C).(-N)^{i} \\ &{}+\sum_{i+j+k=m}\begin{pmatrix}m\\i,j,k\end{pmatrix}CN^{j}C.w_{k}(T,C).(-N)^{i+1} \\ ={}&\sum_{i+j+k=m}\begin{pmatrix}m\\i,j,k\end{pmatrix}CN^{j}C.w_{k+1}(T,C).(-N)^{i} \\ &{}+\sum_{i+j+k=m}\begin{pmatrix}m\\i,j,k\end{pmatrix}CN^{j+1}C.w_{k}(T,C).(-N)^{i} \\ &{}+\sum_{i+j+k=m}\begin{pmatrix}m\\i,j,k\end{pmatrix}CN^{j}C.w_{k}(T,C).(-N)^{i+1} \\ ={}&\sum_{i+j+k=m+1}\begin{pmatrix}m+1\\i,j,k\end{pmatrix}CN^{j}C.w_{k}(T,C).(-N)^{i}. \end{aligned}$$

(2.2) holds for $m+1$, and hence it holds for any $m\in\mathbb {N}$. By (2.2),

$$w_{m+2n-2}(T+N,C)=\sum_{i+j+k=m+2n-2} \begin{pmatrix}m+2n-2\\i,j,k\end{pmatrix}CN^{j}C.w_{k}(T,C).(-N)^{i}. $$

(i) If $\operatorname{max}\{i,j\}\geq n$, then $CN^{j}C=0$ or $N^{i}=0$.
(ii) If $\operatorname{max}\{i,j\}\leq n-1$, then $k\geq m$ and hence $w_{k}(T,C)=0$.

By (i) and (ii), $w_{m+2n-2}(T+N,C)=0$. Therefore $T+N$ is an [$m+2n-2$]-complex symmetric operator with conjugation *C*. □

### Example 2.1

Let *C* be a conjugation given by $C(z_{1}, z_{2}, z_{3}) = (\overline {z_{3}}, \overline{z_{2}}, \overline{z_{1}})$ on $\mathbb {C}^{3}$.

If $T= \left( {\scriptsize\begin{matrix}{} 1&m&m\cr 0&1&0\cr 0&0&1 \end{matrix}} \right)$ on $\mathbb {C}^{3}$, we have $CTC= \left( {\scriptsize\begin{matrix}{} 1&0&0\cr 0&1&0\cr \overline{m}&\overline{m}&1 \end{matrix}} \right)$, then

$$\begin{aligned} &(CTC)^{3}-3(CTC)^{2}.T+3(CTC).T^{2}-T^{3} \\ &\quad =\left ( \begin{matrix} 1&0&0\\ 0&1&0\\ \overline{m}&\overline{m}&1 \end{matrix} \right )^{3}-3\left ( \begin{matrix} 1&0&0\\ 0&1&0\\ \overline{m}&\overline{m}&1 \end{matrix} \right )^{2}\left ( \begin{matrix} 1&m&m\\ 0&1&0\\ 0&0&1 \end{matrix} \right ) \\ &\qquad{}+3\left ( \begin{matrix} 1&0&0\\ 0&1&0\\ \overline{m}&\overline{m}&1 \end{matrix} \right )\left ( \begin{matrix} 1&m&m\\ 0&1&0\\ 0&0&1 \end{matrix} \right )^{2}-\left ( \begin{matrix} 1&m&m\\ 0&1&0\\ 0&0&1 \end{matrix} \right )^{3} \\ &\quad =0. \end{aligned}$$

Hence *T* is a [3]-complex symmetric operator.

On the other hand, since $T = I + N$, where $N= \left( {\scriptsize\begin{matrix}{} 0&m&m\cr 0&0&0\cr 0&0&0 \end{matrix}} \right)$, $N^{2} = 0$, it follows from Theorem 2.4 that *T* is a [3]-complex symmetric operator.
